# National Influenza Vaccination Week — December 7–13, 2014

**Published:** 2014-12-05

The U.S. Department of Health and Human Services, CDC, state and local health departments, and other health agencies will observe National Influenza Vaccination Week December 7–13, 2014, with educational and promotional activities scheduled across the country. The observance was begun in 2005 to highlight the importance of annual influenza vaccination and to foster greater use of influenza vaccine in the months of December, January, and beyond. As of November 14, 2014, approximately 139.7 million doses of 2014–15 seasonal influenza vaccine had been distributed to vaccination providers in the United States (1).

The Advisory Committee on Immunization Practices recommends influenza vaccination for all persons aged ≥6 months (2). Influenza vaccination is especially important for certain persons at higher risk for influenza-related complications. Persons in high-risk groups include children aged <5 years, especially those aged <2 years; persons with certain chronic health conditions, such as heart disease, asthma, and diabetes; pregnant women; and adults aged ≥65 years. In addition, health care personnel are at greater risk for acquiring influenza and can transmit it to their patients (3).

Educational materials, web tools, and CDC’s planned activities for National Influenza Vaccination Week are available at http://www.cdc.gov/flu/nivw/index.htm, whereas general materials regarding influenza vaccination are available at http://www.cdc.gov/flu/freeresources. Additional information and resources for health care professionals are available at http://www.cdc.gov/flu/professionals. End of season vaccination coverage estimates for the 2013–14 influenza season are available at http://www.cdc.gov/flu/fluvaxview.
